# Enhancement of bioactive compounds and biological activities of *Centella asiatica* through ultrasound treatment

**DOI:** 10.1016/j.ultsonch.2023.106353

**Published:** 2023-03-04

**Authors:** Eunjeong Seong, Huijin Heo, Heon Sang Jeong, Hana Lee, Junsoo Lee

**Affiliations:** Department of Food Science and Biotechnology, Chungbuk National University, Cheongju, Chungbuk 28644, Korea

**Keywords:** Ultrasound, Post-harvest treatment, *Centella asiatica*, Elicitation, Myoblasts

## Abstract

*Centella asiatica* possess various health-promoting activities owing to its bioactive compounds such as triterpenes, flavonoids, and vitamins. Ultrasound treatment during the post-harvest process is a good strategy for eliciting secondary metabolite in plants. The present study investigated the effect of ultrasound treatment for different time durations on the bioactive compounds and biological activities of *C. asiatica* leaves. The leaves were treated with ultrasound for 5, 10, and 20 min. Ultrasound elicitation (especially for 10 min) markedly elevated the accumulation of stress markers, leading to enhanced phenolic-triggering enzyme activities. The accumulation of secondary metabolites and antioxidant activities were also significantly improved compared with that in untreated leaves. In addition, ultrasound-treated *C. asiatica* leaves protected myoblasts against H_2_O_2_-induced oxidative stress by regulating reactive oxygen species production, glutathione depletion, and lipid peroxidation. These findings indicate that elicitation using ultrasound can be a simple method for increasing functional compound production and enhancing biological activities in *C. asiatica* leaves.

## Highlights

• *Centella asiatica* leaf is an excellent source of numerous phytochemicals.
• Ultrasound as a postharvest elicitor increased accumulation of bioactive compounds.
• Ultrasound significantly elevated activities of phenolic triggering enzymes.
• *Centella asiatica* can protect myoblast via modulation ROS, GSH, and MDA levels.

## Graphical abstract

**Figure:**
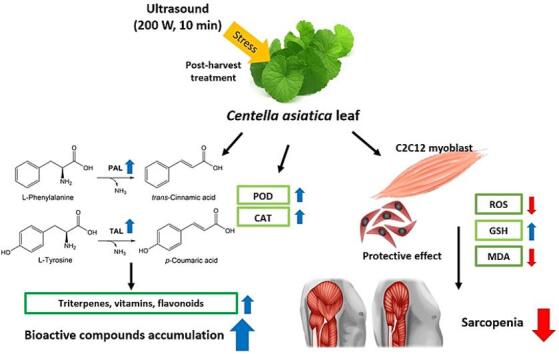

## 1 Introduction

Elicitors are classified as biotic (organic acids, microbes, and hormones) and abiotic (hypoxia, salts, temperature, and light) [1]. Increasing evidence show that the application of elicitors enhances the various secondary metabolites compared with the control levels [2]. These elicitors need to be sustainable, cheap, and generally recognized as safe. Ultrasound technology is widely used in the food industry because it is safe, non-toxic, and eco-friendly [3]. The mechanism underlying the action of ultrasound is the formation of a multiple-bubble system and bubble coalescence process, leading to oxidative burst and the finally inducing a defense system in plants [4]. Previous studies have shown that ultrasound enhances genistein, daidzein, and gamma-aminobutyric acid contents in soybean sprouts and increases resveratrol content in peanut sprouts [5], [6]. In addition, ultrasound treatment triggers membrane ion fluxes and rapidly increases tyrosine ammonia-lyase (TAL) and phenylalanine ammonia-lyase (PAL) activities, followed by increase in phenolic compounds and polyphenol concentrations [7], [8].

*Centella asiatica* (L) Urban is an herb used to prevent or treat diseases in traditional Chinese medicine in Asian countries [9]. *C. asiatica* contains various bioactive compounds, including phenolic compounds, triterpenes, minerals, and vitamins [10]. Based on numerous studies, the triterpenes, including asiaticoside, madecasosside, asiatic acid, and madecassic acid, are believed to be the major bioactive compounds of *C. asiatica* [11]. Previous studies have attributed relieving and therapeutic effects to *C. asiatica* and its bioactive compounds with regard to cardioprotection, wound healing, and neuroprotection [12]. *C. asiatica* alleviates neurological diseases by reducing inflammatory factors, repairing abnormal expression of mitochondria-related proteins, and balancing oxidative stress [13]. Pittella et al. (2009) investigated the total phenolic and flavonoid constituents and antioxidant activities of *C. asiatica* leaves and established a positive correlation between antioxidant and antitumor activities [14]. The ameliorative effect of *C. asiatica* on various diseases may be due to its excellent antioxidant potential.

Skeletal muscle is an important component of the body and constitutes approximately 50% of the body mass [15]. Sarcopenia is a generalized loss of muscle mass and function that occurs in the absence of underlying diseases and is characterized by aging-associated progression [16]. The damage caused is irreversible and destructive and is instigated by oxidative stress in skeletal muscle cells [17]. Oxidative stress is induced by the homeostatic impairment of reactive oxygen species (ROS) and leads to several pathological conditions including obesity, inflammation, and aging [18]. A previous study has confirmed that asiatic acid exhibits antioxidative and antiapoptotic effects by reducing ROS production [19]. Anand et al. (2012) demonstrated that administration of *C. asiatica* extract suppressed physical fatigue by increasing glycogen stores and antioxidant enzymes and decreasing lipid peroxidation in a rat model [20]. Therefore, utilizing *C. asiatica* could be a strategy for the protection of skeletal muscle. However, the accumulation of biological compounds due to post-harvest treatment using ultrasound in *C. asiatica* leaves and their protective effect on oxidative stress in C2C12 myoblasts remain unknown. The present study aimed to determine the effect of ultrasound treatment on the antioxidant capacity, bioactive compounds content, and biological activity of *C. asiatica* leaves.

## 2 Materials and methods

### 2.1 Ultrasound treatment and sample preparation

*C. asiatica* was purchased from a farm (Hapcheon, Korea) and stored in cold room at 4 ℃. Fifty grams of the sample was transferred into an ultrasonic cleaner (WUC-D10H, Daihan Scientific, Korea, 290×240×150 mm, 230v/40 kHz, 400 W) and treated with ultrasound at 200 W and for 5, 10, and 20 min at 25 ℃. After treatment, the ultrasound treated *C. asiatica* was stored at −80 ℃ until further use. For analysis, 3 g of lyophilized *C. asiatica* leaves was subjected to extraction using methanol.

### 2.2 Determination of triterpenes

To confirm the quantities of major four triterpenes including madecassoside, asiaticoside, madecassic acid, and asiatic acid, dried *C. asiatica* leaves were ground into a fine powder. Each leaf (0.1 g) was subjected to extraction using 80% methanol and sonicated for 30 min. The extracts were filtered through a Toyo No. 2 filter paper (Toyo Ltd., Tokyo, Japan), and diluted to 50 mL. The triterpenes were analyzed using HPLC in a Luna C18 column (250×4.6 mm, 5 µm, Phenomenex, Torrance, CA, USA). A fluorescence detector was used at an excitation of wavelength 205 nm. The mobile phase consisted of 0.05% phosphoric acid in water and acetonitrile. Gradient elution was performed under the following conditions: 0–30 min, 20% B; 30–40 min, 100% B; 40–50 min, 20% B. The flow rate was 1.0 mL/min. A UV detector (UV-2075, Jasco) was used at wavelength of 205 nm.

### 2.3 Determination of total flavonoid and total polyphenol contents and antioxidant activities

The total phenolic and total flavonoid contents in *C. asiatica* leaves were measured using the Folin–Ciocalteu colorimetric method and another colorimetric method based on one described in a previous study, respectively [21]. ABTS and DPPH radical scavenging activities and reducing power were determined according to a previously described method [22].

### 2.4 Determination of chlorophyll content

Dried sample (0.05 g) was subjected to extraction using 80% dimethyl sulfoxide and incubated at 65 ℃ for 1 h. The extracts were then centrifuged for 5 min at 10,000× g. The supernatant was assayed using a spectrophotometer (BioTek, Inc., Winooski, VT, USA) at wavelengths of 665 and 648 nm.

Chl a = 14.85 × A_665_ – 5.14 × A_648_.

Chl b = 25.48 × A_648_ – 7.36 × A_665_.

Total chlorophyll = 7.49 × A_665_ + 20.34 × A_648_ (expressed in mg/g dry weight).

### 2.5 Determination of vitamins

Vitamin E and C levels were determined using HPLC according to a previously described methods [23], [24]. Vitamin E was analyzed using normal phase HPLC in a Lichrospher 100 Diol column (250×4.6 mm, 5 μm, Merck, Berlin, Germany) and vitamin C using reverse phase HPLC in a C18 column (250×4.6 mm, 5 μm, Shisheido, Tokyo, Japan), respectively.

### 2.6 Determination of flavonoids

Lyophilized leaves were extracted with methanol containing 10% phosphoric acid (0.1% [v/v]) and the mixture was centrifuged at 1000× g for 5 min. Rutin, catechin, and naringin were quantified using HPLC in a Luna C18 column (250 mm × 4.6 mm, 5 μm, Phenomenex, USA). Water (A) and 5% acetic acid in methanol (B) were used as the mobile phase. Gradient elution was carried out under the following conditions: 0–10 min, 0%–20% B; 10–20 min, 20%-40% B; 20–30 min, 40%–50% B; 30–40 min, 50%–70% B; and 40–50 min, 70%–100% B.

### 2.7 Tyrosine ammonia-lyase (TAL) and phenylalanine ammonia-lyase (PAL) assays

TAL and PAL activities of the ultrasound-treated *C. asiatica* leaves were evaluated as previously described [25]. TAL enzymatic activity was measured by confirming the production of *p*-coumaric acid from the l-tyrosine in the supernatant at 310 nm. PAL enzymatic activity was determined by confirming the production of *trans*-cinnamic acid from l-phenylalanine in the supernatant at 290 nm.

### 2.8 Catalase (CAT) and peroxidase (POD) assays

Ultrasound-treated *C. asiatica* leaves were homogenized in 5 mL phosphate buffer (10 mM, pH 7.4). The extracts were centrifuged (14,240 × g for 15 min) and the supernatant was collected. For the CAT assay, 250 μL of the extract, 2.5 mL of phosphate buffer (10 mM, pH 7.4), and 200 μL of water (100 mM) were mixed in a tube. For the POD assay, 100 μL of the extract, 3 mL of phosphate buffer (0.05 M, pH 6.0), 150 mM of guaiacol, and 200 μL of water (100 mM) were mixed in a tube. Detection was performed using a spectrophotometer at 240 nm and 450 nm, respectively for 30 min. The results were expressed as U/g fresh weight (FW).

### 2.9 Cell culture and cell viability

C2C12 cells were obtained from ATCC (CRL-1772, Manassas, VA, USA). C2C12 myoblasts were seeded in 96-well plates at a density of 5.0×10^4^ cells/mL. After 24 h, the cells were treated with untreated- or ultrasound-treated *C. asiatica* (50 μg/mL). After 2 h, the culture medium was replaced with hydroperoxide (700 μM) and the samples. After 24 h, 20 μL of MTT (5 mg/mL) was added to each well and incubated for 2 h. The supernatant was then removed, and the blue crystal formazan crystals produced in viable cells were dissolved in dimethyl sulfoxide.

### 2.10 Cellular reactive oxygen species (ROS), glutathione (GSH), and lipid peroxidation

ROS production was determined as previously described [26]. C2C12 cells (5.0×10^4^ cells/mL) were seeded in a 96-well black plate. After 24 h, the cells were pre-incubated with the samples for 5 h. The supernatant was then removed, and 10 μM of DCFH-DA with 700 μM hydroperoxide was added to each well at 37 ℃. ROS levels were determined using a fluorescence spectrophotometer (LS-55; Perkin-Elmer, Norwalk, CT, USA). To determine GSH and MDA levels, C2C12 cells were seeded in 6-well plates at a density of 1.5×10^5^ cells/mL. After 24 h, the culture medium was replaced with an FBS-free medium containing extracts. After 4 h, the cells were treated with 700 μM hydroperoxide for 24 h to induce oxidative stress. The cells were then extracted and centrifuged. To measure the GSH levels, 20 µL of the supernatant was added to 180 µL of a mixture containing glutathione reductase, NADPH, and DTNB. The lipid peroxidation level was determined using the thiobarbituric acid reactive substance (TBARS) assay [26].

### 2.11 Statistical analysis

Data are representative of two or three independent experiments and were analyzed using GraphPad Prism software version 5 (GraphPad Software Inc., La Jolla, CA, USA) and SAS version 9.4 (SAS Institute Inc., Cary, NC, USA).

## 3 Results and discussion

### 3.1 Effects of ultrasound treatment on triterpenes accumulation in C. Asiatica leaves

Ultrasound treatment can be used as a post-harvest elicitor to increase the amounts of secondary metabolites [27]. It was reported that elicitors can switch the enzymatic responses to abiotic or biotic stresses, leading to the accumulation of secondary metabolites [28]. The major compounds responsible for bioactivity in *C. asiatica* are two glycosides, madecassoside and asiaticoside, and corresponding two aglycones, madecassic acid and asiatic acid [29]. The present study investigated the changes in the content of the main triterpenes induced by ultrasonic treatment of *C. asiatica* leaves. Table 1 shows that *C. asiatica* leaves contains higher levels of the glycoside form than of the aglycone form. The highest concentration of a secondary metabolite in untreated leaves corresponded to asiaticoside (7.46±0.35 mg/g dry weight), followed by madecassoside (5.59±0.07 mg/g dry weight). Ultrasound treatment increased the amounts of madecassoside, asiaticoside, and madecasic acid in a time-dependent manner. Also, total triterpene content was significantly enhanced at 20 min (19.12±0.08 mg/g dry weight) compared with that in the untreated leaves (15.36±0.21 mg/g dry weight). A previous study showed that ultrasound treatment for 20 min significantly stimulated the secretion of oleanolic acid saponins in marigold hairy root [30]. Puttarak and Panichayupakaranant (2012) reported that the total triterpenes content in *C. asiatica* leaves was 19.5±0.9 mg/g dry weight [31]. Moreover, our results are in conformation with that of a previous study, which demonstrated that the triterpenes accumulated in *C. asiatica* leaves were in the glycoside form rather than the aglycone form [31]. Taken together, our findings indicate that ultrasound treatment could enhance triterpenes levels in *C. asiatica* leaves.

**Table 1:** Triterpene contents in *Centella asiatica.*

Samples	Madecassoside	Asiaticoside	Madecassic acid	Asiatic acid	Total	
	(mg/g DW^1^)					
Raw (untreated)	5.59±0.07^b^	7.46±0.35^c^	0.32±0.02^c^	1.99±0.09^a^	15.36±0.21^c^	
5 min	6.31±0.48^b^	8.40±0.14^b^	0.39±0.02^b^	1.83±0.05^ab^	16.93±0.69^b^	
10 min	7.40±0.19^a^	9.30±0.32^a^	0.44±0.02^b^	1.74±0.06^bc^	18.88±0.59^a^	
20 min	7.50±0.57^a^	9.47±0.07^a^	0.51±0.02^a^	1.64±0.01^c^	19.12±0.08^a^	

All values are means of duplicate, and the mean values in a column followed by different superscript letters are significantly (*p* < 0.05) different (Duncan's multiple range test).

a ^1^ DW = Dry weight.

### 3.2 Effects of ultrasound treatment on bioactive compounds in C. Asiatica leaves

To investigate the effect of ultrasound treatment, we investigated the changes in the phytonutrient components of *C. asiatica*. We found that the polyphenol, flavonoid, vitamin, and chlorophyll contents of *C. asiatica* were greatly altered by ultrasound treatment (Table 2). Total phenolic and total flavonoid constituents were significantly higher in leaves with ultrasound treatment for 5, 10, and 20 min than in untreated leaves. The best time duration for ultrasound treatment was determined to be 20 min, which induced the highest increase of total polyphenol (2255.21±33.35 GAE mg/100 g DW) and total flavonoid contents (2219.80±5.39 CE mg/100 g DW). Additionally, ultrasound treatment impacted the concentration of flavonoid content including that of catechin, naringin, and rutin in *C. asiatica*. The concentration of catechin, naringin, and rutin in ultrasound untreated *C. asiatica* was 0.92±0.11, 0.04±0.00, and 0.35±0.03 g/100 g DW, respectively. Post-harvest treatment of *C. asiatica* for 10 min with ultrasound significantly enhanced the levels of catechin (1.24±0.22 g/100 g DW), naringin (0.07±0.00 g/100 g DW), and rutin (0.45±0.08 g/100 g DW). The increase could be attributed to the triggering of shikimic acid–phenylpropanoid metabolism, resulting in the biosynthesis and accumulation of flavonoids and phenolics [32].

**Table 2:** Functional compounds in *Centella asiatica*.

	Raw (untreated)	5 min	10 min	20 min
Total polyphenol (GAE^2^ mg/100 g DW^1^)	1992.70±18.33^d^	2089.83±20.25^c^	2192.12±21.16^b^	2255.21±33.35^a^
Total flavonoid (CE^3^ mg/100 g DW)	1746.56±32.22^d^	1948.26±31.86^c^	2085.98±54.62^b^	2219.80±5.39^a^

*Flavonoids*				
Catechin (g/100 g DW)	0.92±0.11^a^	1.00±0.09^a^	1.24±0.22^a^	1.16±0.01^a^
Naringin (g/100 g DW)	0.04±0.00^b^	0.05±0.00^ab^	0.07±0.00^a^	0.05±0.01^ab^
Rutin (g/100 g DW)	0.35±0.03^a^	0.39±0.01^a^	0.45±0.08^a^	0.41±0.02^a^

*Vitamins*				
Vitamin C (mg/100 g DW)	44.34±0.43^c^	57.87±7.08^b^	70.43±2.10^a^	59.00±0.27^b^
α-Tocopherol (mg/100 g DW)	13.76±1.18^a^	12.60±0.68^a^	13.57±1.12^a^	13.64±1.09^a^
β-Tocopherol (mg/100 g DW)	0.07±0.03^a^	0.05±0.00^a^	0.06±0.00^a^	0.06±0.01^a^
γ-Tocopherol (mg/100 g DW)	0.25±0.13^a^	0.23±0.01^a^	0.23±0.03^a^	0.24±0.03^a^
γ-Tocotrienol (mg/100 g DW)	0.04±0.01^a^	0.04±0.00^a^	0.05±0.01^a^	0.04±0.01^a^

*Chlorophylls*				
Chlorophyll *a* (mg/g DW)	40.62±6.58^a^	46.01±0.02^a^	47.52±0.31^a^	45.45±2.23^a^
Chlorophyll *b* (mg/g DW)	8.97±1.03^b^	10.49±0.08^ab^	10.73±0.11^a^	10.19±0.53^ab^

All values are the means of duplicate trials, and the mean values in a row followed by different superscript letters are significantly different (p < 0.05) (Duncan's multiple range test).

a ^1^ DW = Dry weight.

b ^2^ GAE = Gallic acid equivalent.

c ^3^ CE = Catechin equivalent.

Plant-derived vitamins such as ascorbic acid and tocopherol act as antioxidants and provide various health benefits ranging from providing basic nutrition to reducing the risk of cancer and chronic diseases [33]. Chlorophylls being the most abundant pigments in plants have huge antioxidant potential [34]. Vitamin C content was markedly enhanced by treatment with ultrasound for 10 min (70.43±2.10 mg/100 g DW) compared with that in untreated leaves (44.34±0.43 mg/100 g DW). Meanwhile, no significant changes were noticed in the vitamin E content of *C. asiatica* after ultrasound treatment. The amounts of chlorophyll *a* and *b* ranged between 40.62 and 47.52 mg/g DW and 8.97–10.73, respectively. Chlorophyll *a* content was not significantly altered, however, Chlorophyll *b* content was significantly increased by ultrasound treatment for 10 min. Our results thus confirm the recent reports demonstrating the capacity of abiotic elicitors to promote the accumulation of functional compounds, such as flavonoids, and vitamins in various plants [25], [35], [36]. Yu et al. (2016) reported that ultrasound treatment enhanced resveratrol contents in peanut sprouts compared with that of the control [6], whereas, Yang et al. (2015) showed that it increased the daidzein, genistein, and gamma-aminobutyric acid contents in soybean sprouts [5]. The reason for the increase of these bioactive compounds could be that ultrasound as a stress-elicitor may have evoked ROS generation. As a defense response, secondary metabolite levels are increased to eliminate the detrimental effects of oxidative damage [37]. Based on these findings, post-harvest treatment with ultrasonication for 10 min might be a efficient way to increase phytochemical accumulation in *C. asiatica*.

### 3.3 Effect of ultrasound treatment on antioxidant capacities in C. Asiatica leaves

**Fig. 1:**
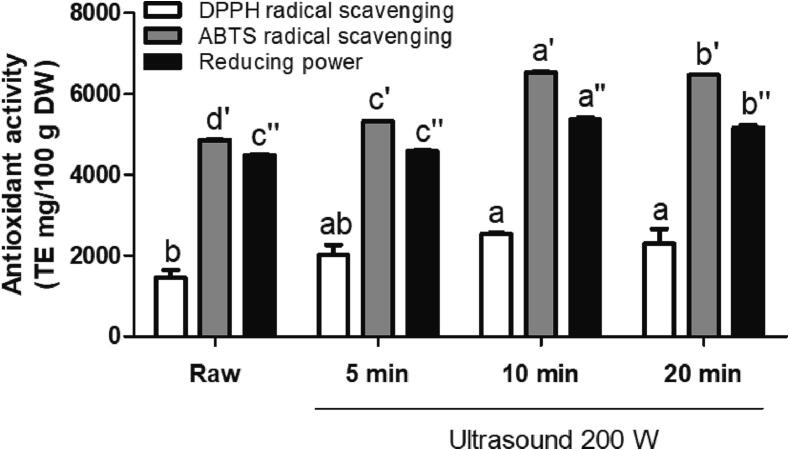
Effect of ultrasound-treated *Centella asiatica* on 1,1-diphenyl-2-picrylhydrazyl (DPPH) radical scavenging activity, (2,2-azino-bis-3-ethylbenzothiazoline-6-sulfonic acid (ABTS) radical scavenging activity, and reducing power. The DPPH and ABTS radical scavenging activity and reducing power are expressed as Trolox equivalent mg/100 g dry weight. Different letters (a,b for DPPH, a′,b′,c′,d’ for ABTS, and a′′,b′′,c′′ for reducing power) above the bars indicate significant differences according to Duncan's test (*p* < 0.05).

The antioxidant activity of ultrasound-treated *C. asiatica* is shown in Fig. 1. Compared with that in the untreated leaves, treatment with ultrasound for 10 min significantly increased the DPPH radical scavenging activity, ABTS radical scavenging activity, and reducing power 1.75-, 1.35-, and 1.20-fold, respectively. Yu et al. (2016) reported that ultrasound-treated romaine lettuce was exhibited a significantly higher DPPH antioxidant activity [38]. Our results are similar to those of Gani et al. (2016), who found that the DPPH and ABTS radical scavenging activities of ultrasonic-treated strawberries increased with treatment time [39]. However, antioxidant activities were slightly reduced after 20 min of ultrasound treatment than at 10 min. This reduction in antioxidant activity may be related to the decrease in flavonoid and vitamin C contents. Generally, when plants are exposed to elicitation conditions, they may activate secondary metabolism as a defense strategy for self-protection. However, plants have thresholds for the quantity of secondary metabolites that they can synthesize. Plant with accumulation of high levels of secondary metabolites stimulate an increase in the levels of enzyme that degrade those metabolites via feedback modulation [40]. González and Nazareno (2011) showed that vitamin C and flavonoids (naringin) exhibit high antioxidant activity [41]. Flavonoids and vitamins are known to exhibit antioxidant activities. A previous study reported that triterpene enrichment in *C. asiatica* extract did not improve its antiradical activity [42]. The triterpenes in *C. asiatica* have various health-promoting effects; however, they may not be directly responsible for their antiradical capacity. Hence, our findings suggest that ultrasound treatment for 10 min would enhance antioxidant activity by increasing the vitamin C and flavonoid contents in *C. asiatica*.

### 3.4 Effect of ultrasound treatment on enzyme activities in C. Asiatica leaves

**Fig. 2:**
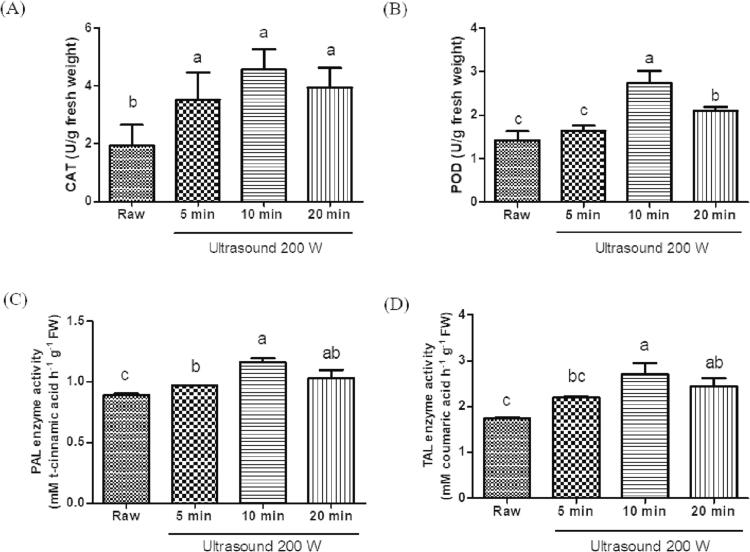
Effect of ultrasound treated *Centella asiatica* on (A) catalase (CAT), (B) peroxidase (POD), and (C) phenylalanine ammonia-lyase (PAL), and (D) tyrosine ammonia-lyase (TAL) activities. Data are presented as the the mean ± standard error (n = 3). Different letters above the bars indicate significant differences at *p* < 0.05.

POD and CAT are stress marker enzymes that play important roles in free radical scavenging [43]. PAL and TAL, which are responsible for the activation of the phenylpropanoid pathway for phenolic biosynthesis, are major markers of plant resistance [44]. TAL and PAL catalyze the conversion of L-tyrosine to p-coumaric acid and L-phenylalanine to *trans*-cinnamic acid, respectively [35]. To the best of our knowledge, no study has yet demonstrated the accumulation of stress markers in *C. asiatica* due to ultrasound elicitation during the post-harvest processing. We found that, compared with that in the untreated leaves, ultrasound treatment significantly enhanced CAT and POD activity in a time-dependent manner (Fig. 2A and B). The highest CAT and POD activity was observed at 10 min. Similarly, compared with that in the untreated leaves, PAL and TAL activities in *C. asiatica* leaves were enhanced 1.30- and 1.55-fold, respectively, after ultrasound treatment for 10 min (Fig. 2C and D). Ampofo and Ngadi (2020) found that ultrasound treatment elicited TAL and PAL activities in bean sprouts [7]. According to a previous study, the disruption of plant tissue leads to H_2_O_2_ accumulation in the cell walls, resulting in the induction of defense-related enzymes such as TAL and PAL for the increase of phenolic compound biosynthesis [45]. Therefore, our findings suggest that ultrasound application during the post-harvest processing of *C. asiatica* enhances the demand for CAT and POD to decompose H_2_O_2_, protect plant cells from oxidative damage, and increase the activity of PAL and TAL to synthesize flavonoids and phenolics.

### 3.5 Cytoprotective effect of ultrasound–treated C. Asiatica leaves against H_2_O_2_-induced oxidative stress in C2C12 myoblasts

**Fig. 3:**
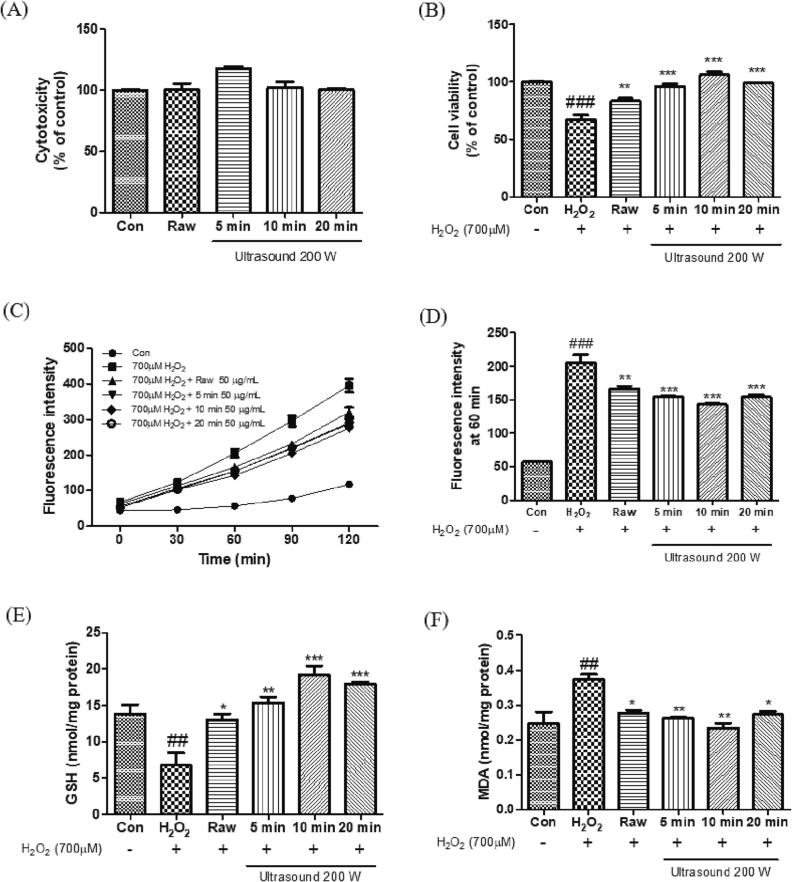
Effect of ultrasound treated *Centella asiatica* extracts (50 μg/mL) on (A) cytotoxicity, (B) protective activity, (C and D) reactive oxygen species production, (E) glutathione depletion, and (F) lipid peroxidation against H_2_O_2_-induced C2C12 cells. Data are presented as the the mean ± standard error (n = 3). ^##^*p* < 0.01 and ^###^*p* < 0.001, versus the control cells; *p < 0.05, ^**^p < 0.01, and ^***^p < 0.001 versus the H_2_O_2_-treated cells. Con, Control; GSH, glutathione; MDA, malondialdehyde.

ROS accumulation is one of the factors that can cause sarcopenia [46]. Antioxidant defense and lipid peroxidation in the body have been suggested as early biomarkers of sarcopenia [47]. In this study, we investigated whether ultrasound-treated *C. asiatica* protects myoblasts against oxidative stress, and we confirmed the protective effect, ROS generation, GSH levels, and lipid peroxidation levels. Treatment with the extract (50 μg/mL) did not affect the cytotoxicity of myoblasts (Fig. 3A). Treatment with hydroperoxide (700 μM) reduced cell viability by 32.7% (Fig. 3B). However, treatment with *C. asiatica* increased cell viability by 24.2 (raw), 42.9 (5 min), 57.9 (10 min), and 47.3% (20 min), compared with that of H_2_O_2_-treated cells. As shown in Fig. 3C – F, myoblasts treated with H_2_O_2_ showed a significant increase in ROS production, GSH depletion, and lipid peroxidation levels compared to that of control cells, whereas treatment with *C. asiatica* markedly decreased the oxidative stress-induced ROS production and GSH depletion. The lipid peroxidation level was reduced depending on the time duration of ultrasound treatment; however, it increased slightly with longer sonication time (20 min). Our results showed that ultrasound treatment for 10 min was the most effective in inverting the increase in ROS generation, GSH depletion, and MDA levels. These increased bioactivities might be attributed to the enhanced phytochemical composition of *C. asiatica* leaves after post-harvest treatment with ultrasound. Triterpenes, flavonoids, and vitamins are well-known to exert antioxidant activity. A previous study reported that supplementation with vitamins C and E alleviated oxidative damage and improved muscle function in aged rodents [48]. Another study showed that *C. asiatica* extract stimulates muscle protein synthesis resulting in the restoration of normal muscle structure and mass [49]. Therefore, these results suggest that ultrasound-treated *C. asiatica* leaves play a crucial role in the protection of myoblasts when treated with ultrasound as the elicitor after harvest.

## 4 Conclusion

In the present study, the changes in the bioactive compounds and biological activities of ultrasound- treated *C. asiatica* leaves were investigated. Ultrasound treatment improved the accumulation of secondary metabolites and antioxidant activities in *C. asiatica* leaves. Ultrasound-treated *C. asiatica* leaves enhanced the protective effect by modulating ROS, GSH, and MDA levels in H_2_O_2_-induced C2C12 cells. Thus, ultrasound treatment can be applied as post-harvest process to stimulate the production of functional compounds in may agricultural products.

## Declaration of Competing Interest

The authors declare that they have no known competing financial interests or personal relationships that could have appeared to influence the work reported in this paper.
